# The utility of repeat Xpert MTB/RIF testing to diagnose tuberculosis in HIV-positive adults with initial negative result

**DOI:** 10.12688/gatesopenres.12815.2

**Published:** 2022-02-25

**Authors:** Yasmeen Hanifa, Katherine L. Fielding, Violet N. Chihota, Lungiswa Adonis, Salome Charalambous, Nicola Foster, Alan Karstaedt, Kerrigan McCarthy, Mark P. Nicol, Nontobeko T. Ndlovu, Edina Sinanovic, Faieza Sahid, Wendy Stevens, Anna Vassall, Gavin J. Churchyard, Alison D. Grant

**Affiliations:** 1 TB Centre, London School of Hygiene & Tropical Medicine, London, UK; 2 The Aurum Institute, Johannesburg, South Africa; 3 School of Public Health, Faculty of Health Sciences, University of the Witwatersrand, Johannesburg, South Africa; 4 Mamelodi Hospital, Pretoria, South Africa; 5 Health Economics Unit, School of Public Health and Family Medicine, University of Cape Town, Cape Town, South Africa; 6 Department of Medicine, Chris Hani Baragwanath Hospital, Johannesburg, South Africa; 7 University of the Witwatersrand, Johannesburg, South Africa; 8 Division of Medical Microbiology, Faculty of Health Sciences, University of Cape Town, Cape Town, South Africa; 9 National Health Laboratory Service, Johannesburg, South Africa; 10 Department of Molecular Medicine and Haematology, School of Pathology, Faculty of Health Sciences, University of the Witwatersrand, Johannesburg, South Africa; 11 Advancing Care and Treatment for TB/HIV, South African Medical Research Council Collaborating Centre for HIV and TB, Johannesburg, South Africa; 12 Africa Health Research Institute, School of Nursing and Public Health, University of KwaZulu-Natal, Durban, South Africa

**Keywords:** Tuberculosis, Diagnostic Test, HIV infection, South Africa

## Abstract

**Background: **Amongst HIV-positive adults in South Africa with initial negative Xpert results, we compared the yield from repeating Xpert MTB/RIF (“Xpert”) on sputum to guideline-recommended investigation for tuberculosis (TB).

**Methods:**  A systematic sample of adults attending for HIV care were enrolled in a cohort exploring TB investigation pathways. This substudy was restricted to those at highest risk of TB (CD4<200 cells/mm ^3^ or unknown) who had a negative initial Xpert result.

At attendance for the Xpert result, a repeat sputum sample was stored, and further investigations facilitated per national guidelines. Participants were reviewed monthly, with reinvestigation if indicated, for at least three months, when sputum and blood were cultured for mycobacteria, and the stored sputum tested using Xpert. We defined TB as “confirmed” if Xpert, line probe assay or *Mycobacterium tuberculosis *culture within six months of enrolment were positive, and “clinical” if TB treatment was started without microbiological confirmation.

**Results: **Amongst 227 participants with an initial negative Xpert result (63% female, median age 37 years, median CD4 count 100 cells/mm ^3^), 28 (12%) participants had TB diagnosed during study follow-up (16 confirmed, 12 clinical); stored sputum tested positive on Xpert in 5/227 (2%). Amongst 27 participants who started TB treatment, the basis was bacteriological confirmation 11/27 (41%); compatible imaging 11/27 (41%); compatible symptoms 2/27 (7%); and unknown 3/27 (11%).

**Conclusions:  **Amongst HIV-positive individuals at high risk of active TB with a negative Xpert result, further investigation using appropriate diagnostic modalities is more likely to lead to TB treatment than immediately repeating sputum for Xpert. TB diagnostic tests with improved sensitivity are needed.

## Introduction

Since 2011 the World Health Organization (WHO) has recommended Xpert MTB/RIF (Xpert; Cepheid, Sunnyvale, CA) as the initial diagnostic test for individuals being investigated for HIV-associated tuberculosis (TB) ^1^. TB diagnosis in people living with HIV (PLHIV) is complicated by the high proportion who are smear-negative and/or have extrapulmonary disease ^2^. Although Xpert has superior sensitivity to sputum microscopy, it is less sensitive than culture, with a pooled sensitivity of 61% for smear-negative, culture-positive TB among PLHIV ^3^.

South Africa replaced smear microscopy with Xpert starting in 2011, for all individuals with symptoms suggesting TB ^4^. Further evaluation of those who are HIV-positive and Xpert-negative comprises clinical reassessment, chest radiograph if available, sputum for mycobacterial culture, and treatment with antibiotic if clinically indicated ^4^. In a South African study of 394 patients investigated for TB (irrespective of presence of symptoms) prior to antiretroviral therapy (ART) initiation, the sensitivity of Xpert for smear-negative, culture-positive TB increased from 43% to 62% when a second sample collected at the first visit was tested ^5^. Mathematical modelling using a decision model from South Africa ^6^ suggested that replacing sputum culture with the cheaper option of a second Xpert would reduce loss to follow-up so 1% more patients would start TB treatment ^7^, and save an estimated US$17.4 million per year ^7^. This model assumed, based on limited data, the same sensitivity for the second Xpert test as for the first ^7^, guidelines would be correctly followed ^7^, and only 1% of those with TB symptoms start TB treatment based on a clinical diagnosis ^6^. The strategy of sending a repeat Xpert for HIV-positive individuals whose initial Xpert result is negative has not been evaluated empirically.

The aim of our study was to describe the diagnostic yield from an immediate repeat Xpert on sputum, compared to sequential further investigation guided by South African recommendations, amongst HIV-positive adults being investigated for TB whose initial Xpert result is negative.

## Methods

This “repeat Xpert” substudy was part of “Xpert for people attending HIV/AIDS care: test or review?” (XPHACTOR), a prospective cohort study evaluating a risk-based algorithm to prioritise Xpert testing amongst adults attending for routine HIV care in South Africa ^8^.

### XPHACTOR study population, recruitment and procedures

XPHACTOR study flow, procedures and algorithm are described in detail in the supplementary material. In summary, we enrolled a systematic sample of adults (aged ≥18 years) attending four HIV clinics in Gauteng province, irrespective of presence of symptoms suggestive of TB, in the XPHACTOR study. Patients taking anti-tuberculosis treatment within the previous three months were excluded. Patients were enrolled into three groups: “on ART” (ART-experienced); “pre-ART” (in HIV care but not taking ART); and “HIV Testing and Counselling (HTC)” (newly-diagnosed HIV-positive). At the time of the study, ART eligibility comprised CD4 ≤350 cells/mm ^3^ or WHO clinical stage ≥3. Research staff screened participants for TB at monthly intervals to three months, using a standardised questionnaire which incorporated the WHO symptom screen (any one of current self-reported cough, fever, weight loss or night sweats, hereafter the WHO tool). A spot sputum sample was collected for Xpert for individuals at *a priori* highest risk of active TB according to the study algorithm, which prioritised testing for those with any of: current cough, fever ≥ 3 weeks, night sweats ≥ 4 weeks, BMI <18.5 kg/m ^2^, CD4 <100 cells/mm ^3^, or weight loss ≥10%; and at enrolment from all in HTC group or pre-ART with CD4 <200 cells/mm ^3^ (Supplementary material, Figure 1).

At enrolment and follow-up visits, participants who submitted an Xpert sample were reviewed within one week, and if Xpert-positive, TB treatment was initiated. If Xpert was negative, research staff repeated WHO symptom screen and facilitated the Xpert-negative algorithm for all who were WHO tool positive, which comprised chest radiograph, spot sputum for TB culture, and/or antibiotic trial as clinically appropriate. The Xpert-negative algorithm was also facilitated, because of *a priori* high risk of active TB, for all pre-ART participants with CD4<200x10 ^6^/l who submitted sputum for immediate Xpert at enrolment to XPHACTOR.

At the three-month visit all participants had sputum and blood cultured for mycobacteria (Bactec MGIT 960 and 9240 systems). We allowed a broad window period around the three-month XPHACTOR main study final visit, until around six months, to maximise follow-up.

Study investigation results were returned to clinic staff, who were responsible for management decisions. Clinic records were reviewed at the end of the study to ascertain any additional relevant investigations (e.g. Xpert, mycobacterial culture and imaging requested as part of routine clinical care) and/or TB diagnoses.

### Repeat Xpert substudy procedures

XPHACTOR participants who were Xpert-negative with i) CD4 count<200 cells/mm ^3^, or ii) new HIV diagnosis (HTC group) were eligible for this substudy, irrespective of presence of WHO tool symptoms; these restrictions aimed to minimise unnecessary testing of individuals at lower risk of active TB. If a participant had more than one negative Xpert result during follow-up, only the first episode was included.

At attendance for Xpert result review, eligible participants were asked for an additional spot sputum sample for “repeat” Xpert, which was frozen at -80°C within 24 hours of collection. All stored samples were thawed and tested with Xpert at the end of the study to evaluate the diagnostic yield that could have been achieved if an immediate repeat Xpert had been sent at the Xpert result review visit. We decided *a priori* not to induce sputum for this substudy in order to reflect what would be achievable in routine practice.

### Definitions

***Repeat Xpert substudy entry and exit dates.*** Repeat Xpert substudy cohort entry date was defined as the date that the Xpert result review was conducted and sputum was collected for storage. Cohort exit date was defined as the last XPHACTOR study visit date.

***TB case definitions.*** “Confirmed” TB was defined as a positive result on i) Xpert (on sputum sample) or ii) line probe assay (LPA) performed on smear-positive or cultured isolate (GenoType MTBDR *plus*, Hain Lifesciences) or iii) *Mycobacterium tuberculosis (Mtb)* culture, from any sample (including stored sputum and those requested by health care providers) collected within six months of XPHACTOR enrolment. Clinical TB was defined as TB treatment started within six months of enrolment ascertained from clinical records, self or family report, or reported in the context of a separate verbal autopsy sub-study, in the absence of microbiological confirmation. Six months was chosen because TB disease evolves gradually ^9, 10^; data from Zimbabwe estimated the mean duration of smear-positivity prior to TB diagnosis amongst HIV-positive adults at 18–33 weeks ^11^.

“Not TB” was defined as absence of criteria for confirmed or clinical TB, and alive at least 3 months (the minimum follow-up period) after enrolment. Participants who did not fulfil the case definitions for TB or “not TB” were deemed to have unclassifiable outcome and excluded from analyses.

Pulmonary and extrapulmonary TB were classified in accordance with WHO definitions ^12^.

We also summarise the basis on which treatment was initiated for those who started TB treatment.

***Radiological definitions.*** “ Probable radiological TB” was defined as presence of any of cavitation, predominantly upper lobe infiltrates, pleural or pericardial effusion, or clear miliary picture on chest radiograph. “ Possible radiological TB” was defined as presence of any of lymphadenopathy (hilar or mediastinal), pulmonary nodules or other infiltrates. Participants with “probable” or “possible” radiological TB features, but without bacteriological confirmation, who started TB treatment within six months of substudy enrolment were assigned as having “clinical” TB.

### Study outcomes

The co-primary outcomes were a) the proportion of participants with a positive repeat Xpert and b) the proportion of participants fulfilling study TB case definitions.

### Statistical methods

Data were analysed using Stata 14 (Stata Corporation, College Station, TX, USA).

We did not undertake formal sample size calculation for this substudy as the sample size was all those eligible from the parent study.

We compared TB diagnoses made by Xpert using the sample stored at substudy enrolment, with all TB diagnoses fulfilling our case definitions during follow-up. We chose this pragmatic comparison because in real life, individuals with smear or Xpert-negative TB have sequential investigation, rather than all tests performed simultaneously. Our research staff facilitated the Xpert-negative algorithm when participants attended for Xpert result review, and therefore investigations are likely to have been initiated faster than in a routine setting. The proportion of TB diagnoses made by Xpert using the stored sputum was compared with TB diagnoses made during follow-up using McNemar’s test.

In a sensitivity analysis restricted to participants who had at least one component of the Xpert-negative algorithm (chest radiograph, sputum for TB culture, or antibiotic trial) within a two-week window of providing the stored repeat Xpert sample, we compared the proportion of TB diagnoses made by Xpert using the stored sputum with TB diagnoses made by the Xpert-negative algorithm using McNemar’s test.

### Ethical approval

The study was approved by the ethics committees at the University of the Witwatersrand (approval # M120343), University of Cape Town (approval # 106/2012), and the London School of Hygiene & Tropical Medicine (approval # 6165). All consenting participants gave written consent or, witnessed verbal consent if unable to read or write. All ethics committees approved the consent form. Principles expressed in the Declaration of Helsinki were followed in the conduct of this research.

## Results

**Figure 1.**
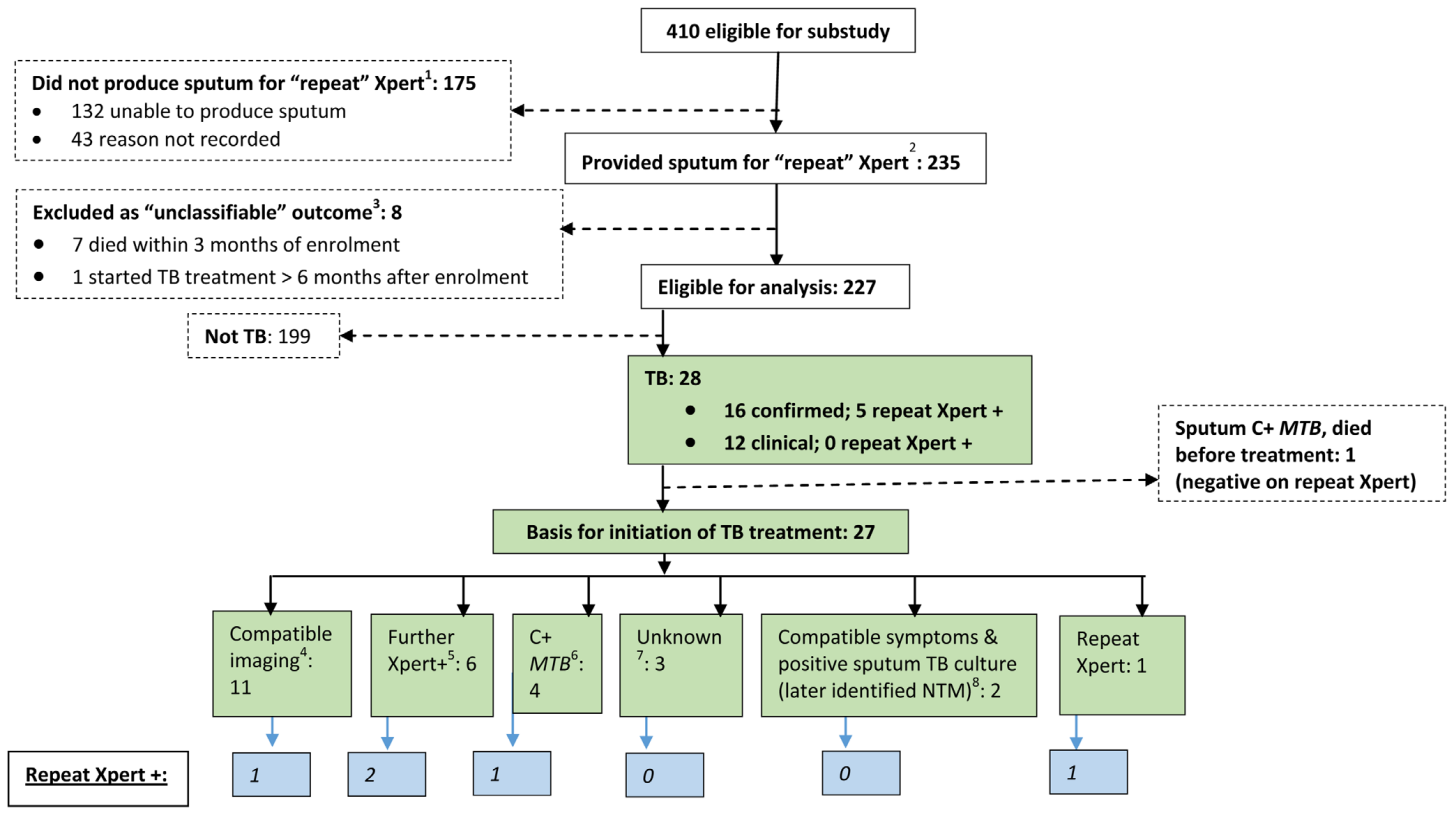
Flow chart of repeat Xpert substudy participants, basis for initiation of TB treatment, and yield from repeat Xpert MTB/RIF. C+ = culture-positive; CXR = chest radiograph; MTB = mycobacterium tuberculosis; NTM = non-tuberculous mycobacteria; USS = ultrasound scan; Xpert+ = sputum Xpert MTB/RIF positive. ^1^ 15/175 who were excluded because they did not produce sputum fulfilled case definitions for TB (clinical TB [9/15], confirmed TB [6/15]) and a further 3/175 had unclassifiable outcome ^2^ For 22/235 participants who provided more than one “repeat” sample (all Xpert-negative) only the result of the first sample and data from the associated review visit were used in the analysis ^3^ All had negative “repeat” Xpert. ^4^ CXR features compatible with “Probable TB” (pleural effusion [4], cavitation and infiltrates [2], miliary TB [1] who had positive repeat Xpert); CXR features compatible with “Possible TB” (2); USS features compatible with TB (Pericardial effusion [1]; abdominal TB [1]). ^5^ Xpert undertaken during study follow-up as part of routine clinical care or for research purposes in accordance with XPHACTOR study algorithm (Supplementary material, Figure 1). ^6^ Samples collected during study follow-up as part of routine clinical care or for research purposes (sputum Mtb culture-positive [3] of whom one had positive repeat Xpert, blood Mtb culture-positive [1]). ^7^ Two identified as having started TB treatment at verbal autopsy. One had M. xenopi identified in sputum culture prior to commencement of empiric TB treatment. ^8^ Started on basis of compatible symptoms and positive sputum culture later identified as M. avium (1) and M. intracellulare (1); both had improvement in symptoms after treatment was initiated.

Between September 2012 and March 2014, 235/410 (57.3%) potentially eligible participants were able to provide a sputum sample, stored for testing at study completion with Xpert ( Figure 1). Eight participants with “unclassifiable” outcome were excluded, leaving 227 participants for analysis.

### Participant characteristics

Characteristics of the 227 substudy participants and comparison with the 175 excluded because they were unable to produce sputum are presented in Table 1. The majority of participants were female (63%), median age was 37 years (interquartile range [IQR] 31,44), median CD4 count was 100 cells/mm ^3^ (IQR 51,147), and 26% had previously been treated for TB. 78/227 (34%) of participants reported a TB symptom, most often cough (23%, 52/227) or weight loss (19%, 43/227) ( Table 1). Amongst the remaining 149/227 (66%) of participants who reported no WHO-tool symptoms at attendance for Xpert result, sputum was collected for repeat Xpert due to *a priori* high risk of active TB because newly-diagnosed HIV-positive (42); pre-ART with CD4 count <200 cells/mm ^3^ (42); CD4 count <100 cells/mm ^3^ (33); on ART with CD4 count 100–199 cells/mm ^3^ (17); BMI <18.5 kg/m ^2^ or weight loss ≥10%, (15). Enrolment to the repeat Xpert study was at median 7 days (IQR 7,8) from collection of the initial sputum sample for Xpert.

**Table 1.** Characteristics of substudy participants (n=227) vs. eligible non-productive of sputum (n=175).

Characteristic	Study participants (N=227)	Did not provide sputum for “repeat” Xpert (N=175)
	N (%)	N (%)
Demographics		
Age, years - Median (IQR)	37 (31-44) (N=226)	36 (30-43)
Female	144 (63.4%)	109 (62.3%)
Black African	222 (97.8%)	175 (100%)
Participant category		
On ART	99 (43.6%)	67 (38.3%)
Pre-ART	75 (33.0%)	60 (34.3%)
HTC	53 (23.4%)	48 (27.4%)
HIV/TB history		
Previous TB treatment	59 (26.0%)	37 (21.1%)
Ever had IPT	18 (7.9%)	6 (3.4%)
Ever had CPT	122 (53.7%)	84 (48.0%)
BMI/CD4 when immediate Xpert was requested		
BMI, kg/m ^2^ - Median (IQR)	23.3 (20.1-27.4) (N=226)	23.4 (20.1-28.1)
CD4 ^1^, cells/mm ^4^ - Median (IQR)	100 (51-147) (N=188)	113 (56-169) (N=148)
WHO tool symptoms when sample for “repeat” Xpert was requested		
WHO-positive	78 (34.4%)	44 (25.1%)
Cough	52 (22.9%)	23 (13.1%)
Weight loss	43 (18.9%)	32 (18.3%)
Night sweats	16 (7.0%)	9 (5.1%)
Fever	7 (3.1%)	2 (1.1%)
TB diagnoses over 6 months follow-up		
Total	28 (12.3%)	18 (10.3%)
Confirmed TB	16 (7.1%)	7 (4.0%)
Clinical TB	12 (5.3%)	11 (6.3%)
Follow-up		
**Time from XPHACTOR** **enrolment to 3-month”** **study visit, days** - Median (IQR)	84 (84,95) (N=220)	86 (84,106) (N=169)

IPT= Isoniazid preventive therapy; BMI = body mass index; CPT= Cotrimoxazole preventive therapy; HTC= Enrolled from HIV testing and counselling service; WHO positive = self-report of any of current cough, fever, night sweats or unintentional weight loss. ^1^ Most recent clinic CD4 cell count when participant attended for Xpert result review. CD4 available for 188/227 participants enrolled (99/99 on ART, 75/75 pre-ART, 14/54 HTC); and 148/175 who did not provide sputum for repeat Xpert” (67/67 on ART, 60/60 pre-ART, 21/48 HTC)

### Tuberculosis diagnoses

12% (28/227) of substudy participants fulfilled case definitions for TB, of which 16 were confirmed and 12 were clinical ( Table 2). One participant died before TB treatment could be commenced, and for one, the treatment start date was unknown. The remaining 26 started TB treatment at a median 49 days (IQR 0,108) after substudy entry. The range for time from substudy entry to earliest of positive TB investigation (including chest radiograph) or date TB treatment was started (amongst all fulfilling our case definitions for TB) was 0–118 days. 24% (19/78) participants who were WHO tool positive when the sample for stored repeat Xpert was collected fulfilled TB case definitions (confirmed 11, clinical 8).

**Table 2.** Basis for TB diagnoses in repeat Xpert substudy (N=28).

Characteristic	Participants diagnosed with TB N=28 N (%)
Case definition	
** Bacteriologically confirmed TB:**	**16 (57%)**
Sputum Xpert positive ^1^	6 (21%)
Sputum *Mtb*culture positive	4 (14%)
Sputum both Xpert and *Mtb* culture-positive	4 (14%)
Blood *Mtb* culture-positive	1 (4%)
Pleural fluid cultured isolate LPA-positive	1 (4%)
** Clinical TB:**	**12 (43%)**
Site of TB	
Pulmonary TB only	18 (64%)
Extrapulmonary TB only ^2^	5 (18%)
Both pulmonary and extrapulmonary TB ^3^	2 (7%)
Not recorded	3 (11%)

LPA = line probe assay ^1^ Includes two participants for whom bacteriological confirmation was provided by stored sputum which was Xpert-positive; one of whom started treatment based on this result, and the other had already started TB treatment because of compatible chest radiograph (miliary TB). ^2 ^ Pleural effusion (3); positive mycobacterial blood culture (1); pericardial effusion (1) ^3^ Compatible abdominal ultrasound and sputum *Mtb* culture-positive (1), pleural effusion and sputum Xpert positive (1)

***Basis for commencement of TB treatment (n=27).*** Thirteen participants started treatment based on positive mycobacteriology. Amongst these, eleven started treatment because of a bacteriologically-confirmed TB result (Xpert [7, of which one was the stored repeat Xpert sample]; *Mtb* isolated from sputum [3] or blood [1]). A further two participants started treatment because of compatible symptoms and positive sputum mycobacterial culture (later identified as non-tuberculous mycobacteria [NTM]) with symptomatic improvement on standard TB treatment ( Figure 1).

Eleven participants started TB treatment because of compatible imaging. Nine had compatible chest radiographs, of whom four were subsequently bacteriologically confirmed (Xpert 2, pleural fluid cultured isolate LPA-positive 1, positive *Mtb* sputum culture 1). Two participants started treatment based on ultrasound scans, one compatible with abdominal TB (subsequently confirmed by positive *Mtb* from sputum culture); and the other showing pericardial effusion. The basis for starting TB treatment was not clear for the remaining three participants ( Figure 1).

**Figure 2.**
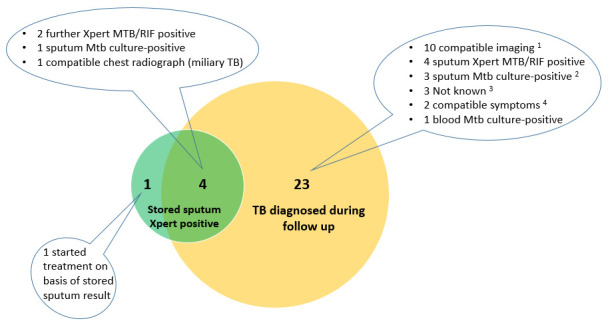
Number of participants diagnosed with TB by “repeat” Xpert vs. number diagnosed during follow-up (N=28). CXR = chest radiograph; USS = ultrasound scan ^**1**^ CXR features compatible with “Probable TB” (pleural effusion [4], cavitation and infiltrates [2]); CXR features compatible with “Possible TB” (2); USS features compatible with TB (Pericardial effusion [1]; abdominal TB [1]) ^2^ One participant died before treatment commenced ^3^ Two identified as having started TB treatment at verbal autopsy. One had *M. xenopi* identified in sputum culture prior to commencement of empiric TB treatment. ^4^ Started on basis of compatible symptoms and positive sputum culture later identified as *M. avium* (1) and *M. intracellulare* (1); both had improvement in symptoms after treatment was initiated.

***Diagnoses made by repeat Xpert on stored sputum samples.*** 5/227 (2.2%) had a positive repeat Xpert. The sensitivity of repeat Xpert was 31.3% (5/16; 95% CI 11.0-58.7%) for the primary outcome of confirmed TB and 18% (5/28, 95% CI: 6.1%-36.9%) for the secondary outcome of confirmed / clinical TB combined) ( Figure 2). In a matched analysis the odds of TB diagnosis was much greater by other modalities during follow-up than by the repeat Xpert, odds ratio 24.0 (95% CI: 3.9–986.9; p<0.0001, McNemar’s test). Amongst the five participants with positive repeat Xpert, three were in the pre-ART group, and two in the on ART group. We were unable to undertake multivariable analysis to look at independent predictors of positive repeat Xpert on the stored sample because only five were positive.

In a sensitivity analysis restricted to 123 participants who had at least one component of the Xpert-negative algorithm within a two-week window of providing the stored repeat Xpert sample, 23 participants fulfilled our TB case definitions (13/23 confirmed, 10/23 clinical). The stored sputum sample was positive by Xpert for four participants (sensitivity of repeat Xpert for confirmed and clinical TB combined 17% [4/23]; for sputum culture-confirmed TB 20% [1/5]). Ten participants started TB treatment because of evaluation by the Xpert-negative algorithm (four confirmed, six clinical), of whom two also had positive stored repeat Xpert. Eleven other participants fulfilled TB case definitions during study follow-up (eight confirmed, three clinical). We did a matched analysis, classifying as “not TB” for the purpose of this analysis, 11 participants who fulfilled our TB case definitions but were not identified by either the Xpert-negative algorithm or stored repeat Xpert. The odds of TB diagnosis by the Xpert-negative algorithm was greater than by repeat Xpert, but did not attain statistical significance, odds ratio 4.0 (95% CI: 0.8–38.7; p=0.11, McNemar’s test).

The participant who started TB treatment solely based on the stored repeat Xpert sample was in the pre-ART group with a CD4 cell count of 113 cells/mm ^3^ at substudy enrolment, had no previous history of TB treatment, and had a five week history of cough and fever when the initial sputum sample for Xpert was collected. The sputum culture, the only component of the Xpert-negative algorithm arranged at the Xpert review visit, was contaminated. This participant initiated ART on the day of entry to the substudy, was WHO-tool negative at all subsequent study visits, and had negative sputum and blood for mycobacterial culture at the 3-month visit. The remaining four participants with positive stored repeat Xpert sample started TB treatment before the stored sample was processed, based on further evaluation during follow-up: sputum Xpert-positive (2, one with rifampicin resistance); sputum *Mtb* culture-positive (1); and compatible chest radiograph (1).

### Investigations performed for participants during substudy follow-up

Figure 2 in the supplementary material summarises all investigations undertaken for TB during substudy follow-up, aside from 3-month visit mycobacterial cultures and Xpert on stored sputum samples. As part of routine care or facilitated by research staff for the Xpert-negative algorithm, 97/227 (43%) had a chest radiograph (38/97 [39%] fulfilled criteria for radiological TB), and 100/227 (44%) had mycobacterial culture on sputum (3/100 [3%] *Mtb* positive). 34/227 (15%) of participants were prescribed an antibiotic trial at the Xpert result review, and 14/21 (67%) of those reviewed reported resolution of symptoms.

89 participants submitted sputum specimens for Xpert as part of routine care or because they fulfilled XPHACTOR algorithm criteria at monthly follow-up visits, for whom 6/89 (7%) were positive. An additional four participants had positive Xpert, of which three were stored sputum samples for repeat Xpert (bacterial confirmation provided solely by stored sample [2], also positive *Mtb* sputum culture [1]), and one was collected after the 3-month visit ( Table 2).

The mycobacterial cultures performed routinely at the 3-month visit yielded *Mtb* isolates in 2% (5/219) of sputum and 0/220 blood samples.

## Discussion

Among HIV-positive individuals at high risk of active TB, with a negative sputum Xpert result, very few TB diagnoses would have been made in this study by immediately repeating Xpert. We limited our study to HIV-positive individuals at highest risk of active TB, i.e. those who were WHO tool positive with CD4<200x10 ^6^/l, or pre-ART with CD4<200x10 ^6^/l, or newly diagnosed, of whom some were asymptomatic, to minimise unnecessary testing of individuals at lower risk of active TB. The low yield from repeat Xpert in those with negative initial Xpert is likely due to paucibacillary or subclinical disease which is more likely in asymptomatic individuals, or extrapulmonary disease. These TB diagnoses may be better identified by alternative diagnostic modalities, such as chest radiography. A South African study of patients with sputum screened for TB by Xpert and mycobacterial culture prior to ART initiation, using a gold standard of culture-confirmed TB (N=85), found that those who were Xpert-negative had higher CD4 cell counts and lower viral loads than those who were Xpert-positive ^13^. We did not have enough positive stored Xpert results to undertake a similar analysis.

Our study illustrates the realities of implementing the test negative algorithm in HIV-positive individuals. Despite research staff facilitating the algorithm, less than half (100/227) of participants produced sputum for mycobacterial culture during follow-up ( *vs.* 100% assumed by Schnippel) ^7^. We found sensitivity of the repeat Xpert was only 18% (5/28) for all TB or 31% (5/16) for bacteriologically-confirmed TB *vs.* 79% assumed by Schnippel ^6, 7^. Data from South Africa demonstrate poor adherence in routine care settings to TB diagnostic algorithms amongst HIV-positive individuals with initial negative Xpert test ^14, 15^. The aforementioned model ^6, 7^ assumes 1% of patients with TB symptoms start TB treatment based on a clinical diagnosis, but we found this to be far greater; and the model does not consider extrapulmonary TB (one-fifth of our participants diagnosed with TB had only extrapulmonary disease). An economic evaluation of repeat sputum Xpert *vs.* the Xpert-negative algorithm for HIV-positive individuals using assumptions that are more realistic is needed.

Evaluation of the 2007 WHO algorithm for smear-negative TB (comprising chest radiograph, single sputum for mycobacterial culture, and antibiotic trial), in HIV-positive individuals being investigated for TB in Cambodia ^16^, against a gold standard of culture-confirmed TB based on multiple specimens, demonstrated sensitivity of 60% ^16^. Sensitivity of this algorithm is imperfect, and there is a risk of overtreatment when only clinical-radiological features are used to start TB treatment. 40% (11/27) of our study participants who started TB treatment did so because of compatible imaging, of whom almost half were subsequently bacteriologically confirmed, highlighting its value to support rapid initiation of TB treatment. Our findings are in accord with data from the XTEND trial, which found that compatible chest radiograph was the main reason for initiating empiric TB treatment in a cohort of patients investigated for TB in primary care in South Africa, amongst whom microbiological confirmation was subsequently obtained for 13% ^17^. South African national guidelines now recommend chest radiography for all individuals with symptoms suggestive of TB who cannot produce a sputum sample, but limited access to radiography facilities may limit implementation ^4^. Amongst our study participants who provided sputum for mycobacterial culture prior to their 3-month visit there was a low yield of *Mtb* (3/100 [3%]), and the yield from further Xpert during follow-up was 7% (6/89), representing just over half (9/16) of all confirmed TB diagnoses. Our findings highlight the need for more sensitive diagnostic tests, and for repeating TB investigation using all available modalities, in HIV-positive individuals with initial negative sputum test result who remain symptomatic or have advanced immunosuppression. 

Our study has some limitations. In the parent XPHACTOR study, Xpert testing was prioritised in people with BMI<18.5kg/m ^2^ or CD4<100, those newly diagnosed HIV-positive or pre-ART with CD4<200, as well as those with TB symptoms ^8^. Thus the population in this substudy did not all have classic “TB symptoms” at the time of collection of either initial or repeat sputum samples for testing with Xpert. These individuals may have had paucibacillary or subclinical disease which would not have been detected by Xpert. However, TB prevalence in our substudy population was high, and we anticipate our results to be relevant at least to these high-risk groups. Our results are also relevant to efforts to improve TB case finding among asymptomatic HIV-positive people ^18, 19^. We froze all our raw sputum samples within 24 hours of collection, and all were thawed and tested within 6 months of collection, in line with other studies ^20^. We assumed that all participants starting TB treatment or with a sample which was bacteriologically confirmed collected within six months of enrolment were likely to have had active TB at enrolment, regardless of whether it was diagnosable using sputum based tests at the time of enrolment. In fact, our study participants who started TB treatment commenced within a median of seven weeks from collection of the “repeat” Xpert sample. Some sputum samples for mycobacterial culture and chest radiographs were taken at an interval after participants returned for their initial Xpert test result, reflecting real-life investigation practice; we cannot be certain of the same result if they had been performed at the same time as sample collection for repeat Xpert. However, our findings suggest that following the Xpert-negative algorithm is more likely to lead to TB diagnosis than immediate repeat Xpert test.

Strengths of our study include systematic evaluation of participants and longitudinal follow-up which minimised the number of TB diagnoses missed, and the pragmatic nature of the study which reflected as far as possible real-life conditions, albeit with optimised implementation of TB diagnostic algorithms.

## Conclusions

Amongst ambulatory HIV-positive individuals at high risk of active TB, if an initial Xpert is negative, the Xpert-negative pathway should be implemented and there should be a low threshold for investigating those who remain at high risk using all clinically appropriate diagnostic modalities. In addition, those for whom no TB diagnosis is made must be made aware of the importance of returning for review if symptoms persist or recur. Our findings do not support sending an immediate repeat Xpert and highlight the need for more sensitive diagnostic tests capable of detecting pulmonary and extrapulmonary TB.

## Data availability

The XPHACTOR “Investigating TB if initial Xpert is negative” dataset, which includes data underlying this substudy, has been uploaded to the LSHTM Data Compass repository: https://doi.org/10.17037/DATA.284 ^21^.

The reader will need to request the dataset from LSHTM (request access is provided within the data record) with a brief summary of how the dataset will be utilised. On request, a data sharing agreement will be made available which will first need to be signed, prior to provision of the dataset. This enables LSHTM to confirm that the reader is using the data for HIV or TB-related research, which is required because study participants consented to use of their data for HIV or TB-related research only.

The data is shared under a Data Sharing Agreement license (see above).

The study team wish to avoid unnecessary barriers to access and will seek to respond to data requests as quickly as possible.

## Supplementary material

Supplementary material: XPHACTOR study flow, procedures and algorithm.

Click here to access the data.
