# Supplementary material for: The utility of repeat Xpert MTB/RIF testing to diagnose tuberculosis in HIV-positive adults with initial negative result
Source: Gates Open Res. 2022 Feb 25;2:22. Originally published 2018 Apr 26. [Version 2] doi: 10.12688/gatesopenres.12815.2 (PMC10495190; doi:10.12688/gatesopenres.12815.2)
Supplement: Peer review history

# Peer review: Reviewer response for version 2

**DOI:** 10.21956/gatesopenres.14812.r34523

**Published:** 2023-08-18

**Authors:** Rohit Sarin

**Affiliations:** 1 National Institute of Tuberculosis and Respiratory Diseases, New Delhi, Delhi, India

In view of the high sensitivity of the Xpert MTB/Rif a single test is sufficient to draw a conclusion and repeat test will not be useful. However, the quality of sputum sample should be good in the first sample. If not or if only saliva tested then a repeat sample will be of additional value. This can be included in the article.

Is the work clearly and accurately presented and does it cite the current literature?

Yes

If applicable, is the statistical analysis and its interpretation appropriate?

I cannot comment. A qualified statistician is required.

Are all the source data underlying the results available to ensure full reproducibility?

Yes

Is the study design appropriate and is the work technically sound?

Partly

Are the conclusions drawn adequately supported by the results?

Yes

Are sufficient details of methods and analysis provided to allow replication by others?

Partly

Reviewer Expertise:

infectious diseases

I confirm that I have read this submission and believe that I have an appropriate level of expertise to confirm that it is of an acceptable scientific standard.

# Peer review: Reviewer response for version 1

**DOI:** 10.21956/gatesopenres.13882.r26494

**Published:** 2018-06-01

**Authors:** Tow Keang Lim

**Affiliations:** 1 Division of Respiratory and Critical Care Medicine, National University Hospital, Singapore, Singapore

This study examined the sensitivity of repeating an expectorated sputum specimen for Xpert testing for TB in a group of high risk immuno-compromised patients  who had returned negative tests initially. negative tests. The repeat Xpert test had lower than expected  sensitivity (~20%) and contributed very little to treatment timeliness. However the overall incidence of TB was low and it was mostly pauci-bacillary disease. These results many not be applicable to patients with higher disease burden or for induced sputa.

Is the work clearly and accurately presented and does it cite the current literature?

Yes

If applicable, is the statistical analysis and its interpretation appropriate?

Yes

Are all the source data underlying the results available to ensure full reproducibility?

Yes

Is the study design appropriate and is the work technically sound?

Yes

Are the conclusions drawn adequately supported by the results?

Yes

Are sufficient details of methods and analysis provided to allow replication by others?

Yes

Reviewer Expertise:

NA

I confirm that I have read this submission and believe that I have an appropriate level of expertise to confirm that it is of an acceptable scientific standard.

# Peer review: Reviewer response for version 1

**DOI:** 10.21956/gatesopenres.13882.r26447

**Published:** 2018-05-31

**Authors:** Colleen F. Hanrahan

**Affiliations:** 1 Department of Epidemiology, Johns Hopkins Bloomberg School of Public Health, Baltimore, MD, USA

Overall

- This study provides some interesting data on how useful a 2nd Xpert is to follow-up on those initially negative by Xpert. The addition of LAM to this study needs to be clearly and explicitly explained. Currently it reads as a very extraneous add-on.
- There are too many tables/figures for the amount of actual data. I find the presentation of data currently to be excessive and unnecessarily complicated and duplicative.

Introduction:

- Aim is awkwardly stated. Possibly reword with study population at the end, and try to make it more succinct.
- LAM needs to be included as an aim from the start. Otherwise it feels like a very tangential add on.

Methods:

- I would like a formal definition of the primary outcome and any secondary outcomes. They are probably in there, but not explicitly stated enough.
- I think a flow diagram of testing would be helpful to flesh out the methods.
- It's not clear to me where LAM fits in. An Xpert negative pt is re-screened for symptoms, has a chest x-ray and sputum for culture, and/or antibiotic trial is given. But then suddenly under statistical methods, you mention LAM for the first time. LAM is not part of the SA algorithm. We need to know when this was done, and even why it is relevant to this study.

Results:

- Not sure why 1 person who started TB treatment >6 months after enrollment would be excluded? This gets back to my previous point that you need to explicitly state the primary outcome, so we can understand why you would have excluded these 7 individuals.
- I'm confused by figure 1. What i'd like to know is what is the additional yield of Xpert, but I can't tell that from this figure. The way it looks is that all the people who had confirmed TB were not diagnosed with clinical TB. However, I can't believe that is true. I would prefer to see who was diagnosed how up front  (clinical, microbiological) and who wasn't ("not tb"). Then from those boxes, show us how many in each were Xpert positive from the 2nd sputum.
- I'm confused by the section on TB diagnosis. Those diagnosed by Xpert don't appear to be by the 2nd Xpert that was taken and stored, because lower down you detail those individuals. But a 2nd Xpert is not part of the SA algorithm.  Something needs to be done to make this clearer.
- Table 2 confuses me for the same reason that figure 1 confuses me.
- If you change figure 1 as suggested above, then you don't need a venn diagram. That diagram conveys very little information.
- Figure 3 is totally confusing because people can be in more than one of the top boxes...I'm not sure what this adds, particularly how it speaks to your primary outcome.

Discussion:

- Although the authors note that their population was not limited to symptomatic individuals as a potential weakness, they do not explicitly state that an asymptomatic population is likely to have paucibacillary or subclinical disease, for which Xpert would not have good sensitivity. The SA algorithm for TB investigation starts with a positive symptom screen, not merely for being HIV positive with a low CD4 despite having no symptoms. So these findings are not necessarily generalizeable to actual practice.
- I would also note that the prevalence of clinical diagnosis is not necessarily generalizeable. Its unclear the level of care that was given to these participants- was this routine care available in a primary public health clinic, or is this specialized care as part of a research study? These details could help the reader evaluate how generalizeable the findings are.

Conclusions:

- The reference to the "Xpert negative pathway" is vague - authors should be specific about what kinds of followup are most useful. Authors should also include something about LAM.

Is the work clearly and accurately presented and does it cite the current literature?

Yes

If applicable, is the statistical analysis and its interpretation appropriate?

Yes

Are all the source data underlying the results available to ensure full reproducibility?

Yes

Is the study design appropriate and is the work technically sound?

Yes

Are the conclusions drawn adequately supported by the results?

Yes

Are sufficient details of methods and analysis provided to allow replication by others?

Partly

Reviewer Expertise:

NA

I confirm that I have read this submission and believe that I have an appropriate level of expertise to confirm that it is of an acceptable scientific standard, however I have significant reservations, as outlined above.

# Response

**Published:** 2022-01-17

**Authors:** YASMEEN HANIFA

**Affiliations:** London School of Hygiene & Tropical Medicine, UK

Dear Dr Hanrahan,

 Thank you very much for your detailed comments and suggestions to improve our manuscript which we found very helpful, and apologies for the delay in responding. I have made changes in accordance with your comments

 Please find below point by point responses to your comments. Your comments are italicized and our responses are in normal font, with bold text to indicate revisions to the manuscript

 Best wishes,

 Yasmeen Hanifa

 ********************

***1. Overall***

- *This study provides some interesting data on how useful a 2nd Xpert is to follow-up on those initially negative by Xpert. The addition of LAM to this study needs to be clearly and explicitly explained. Currently it reads as a very extraneous add-on.*

**Response:** We have removed the LAM data from this manuscript.

- *There are too many tables/figures for the amount of actual data. I find the presentation of data* *currently to be excessive and unnecessarily complicated and duplicative.*

**Response: **We have combined Figures 1 & information from Figure 2 (new Figure 1), moved the data summarising the basis upon which TB treatment was commenced from table 2 to the new Figure 1 and moved Figure 3 to the supplementary material (new Figure 2 in supplementary material). This leaves two figures and two tables in the manuscript.

 ********************

***2. Introduction:***

- *Aim is awkwardly stated. Possibly reword with study population at the end, and try to make it more succinct.*

**Response:** We have amended the text as per your recommendation. The text reads as follows:

**The aim of our study was to describe the diagnostic yield from an immediate repeat Xpert on sputum, compared to sequential further investigation guided by South African recommendations, amongst HIV-positive adults being investigated for TB whose initial Xpert result is negative.**

- *LAM needs to be included as an aim from the start. Otherwise it feels like a very tangential add on.*

**Response:** We have removed the LAM data from this manuscript.

 ********************

***3. Methods:***

- *I would like a formal definition of the primary outcome and any secondary outcomes. They are probably in there, but not explicitly stated enough.*

**Response:** We have amended the text as per your recommendation. The text reads as follows:

**Study outcomes**

 The co-primary outcomes were a) the proportion of participants with a positive repeat Xpert and b) the proportion of participants fulfilling study TB case definitions **.**

- *I think a flow diagram of testing would be helpful to flesh out the methods.*

**Response:** Figure 1 in the supplementary material describes the flow of the XPHACTOR study and this has been amended to more clearly detail the testing for the repeat Xpert substudy.

- *It's not clear to me where LAM fits in. An Xpert negative pt is re-screened for symptoms, has a chest x-ray and sputum for culture, and/or antibiotic trial is given. But then suddenly under statistical methods, you mention LAM for the first time. LAM is not part of the SA algorithm. We need to know when this was done, and even why it is relevant to this study.*

**Response:** We have removed the LAM data from this manuscript.

 ********************

***4. Results:***

- *Not sure why 1 person who started TB treatment >6 months after enrollment would be excluded?* *This gets back to my previous point that you need to explicitly state the primary outcome, so we can understand why you would have excluded these 7 individuals.*

**Response:** We excluded in total eight individuals from our analysis who did not fulfil the case definitions for TB or “not TB”. The rationale for this is detailed in the section headed “TB case definitions”. We deemed these individuals to have unclassifiable outcome and excluded them from all analyses. The primary outcome is now explicitly stated in the methods section as per your recommendation. 

- *I'm confused by figure 1. What i'd like to know is what is the additional yield of Xpert, but I can't tell that from this figure. The way it looks is that all the people who had confirmed TB were not diagnosed with clinical TB. However, I can't believe that is true. I would prefer to see who was diagnosed how up front (clinical, microbiological) and who wasn't ("not tb"). Then from those boxes, show us how many in each were Xpert positive from the 2nd sputum.*

**Response:** Figure 1 has been modified to detail the additional yield of Xpert by combining the information from Figure 2 as per your recommendation. It now clearly details the basis upon which patients actually commenced TB treatment

- *I'm confused by the section on TB diagnosis. Those diagnosed by Xpert don't appear to be by the 2nd Xpert that was taken and stored, because lower down you detail those individuals. But a 2 ^nd^ Xpert is not part of the SA algorithm. Something needs to be done to make this clearer.*

**Response:** Further Xpert investigation was performed during study follow-up both for research purposes in accordance with the XPHACTOR study algorithm (described in the methods and in the supplementary material) and also during routine clinical care. Therefore, participants may have submitted a number of sputum samples for Xpert during the course of the study, and a positive result from any sample was used to assign the TB case definition. This has been made clearer in the footnotes for new Figure 1. The main study methods in the supplementary material describe how we reviewed clinic records at the end of the study to ascertain additional relevant investigations (including TB investigation performed as part of routine clinical care) and/or TB diagnoses.

 We have amended the methods section to clarify this. The text reads as follows:

**Investigation results were returned to clinic staff, who were responsible for management decisions. Clinic records were reviewed at the end of the study to ascertain any additional relevant investigations (e.g. Xpert, mycobacterial culture and imaging requested as part of routine clinical care) and/or TB diagnoses.**

- *Table 2 confuses me for the same reason that figure 1 confuses me.*

**Response: **Table 2 has been simplified to report how case definitions were fulfilled and the site of TB; the summary of the basis upon which TB treatment was initiated has been moved to new Figure 1.

- *If you change figure 1 as suggested above, then you don't need a venn diagram. That diagram conveys very little information.*

**Response:** We have changed Figure 1 as per your recommendation. We think that the Venn diagram conveys the key study result at a glance and complements new Figure 1, particularly by showing graphically the overlap between individuals identified by repeat Xpert vs. the conventional algorithm. We would therefore prefer to retain it, if necessary in the supplementary material.

 We have also amended the results section to clarify the basis for initiating TB treatment. The text reads as follows:

**Thirteen participants started treatment based on positive mycobacteriology. Amongst these, eleven started treatment because of a bacteriologically-confirmed TB result (Xpert [7, of which one was the stored repeat Xpert sample]; *Mtb *isolated from sputum [3] or blood [1]). A further two participants started treatment because of compatible symptoms and positive sputum mycobacterial culture (later identified as non-tuberculous mycobacteria [NTM]) with symptomatic improvement on standard TB treatment (Figure 1).**

- *Figure 3 is totally confusing because people can be in more than one of the top boxes...I'm not sure what this adds, particularly how it speaks to your primary outcome.*

**Response:** Although it does not speak to our primary outcome Figure 3 describes the volume and detail of investigation for TB that study participants underwent. As participants had multiple investigations they were included in more than one of the top boxes. This figure has been moved to the online supplement (Supplementary material, Figure 2) and renamed “Investigations performed for participants from repeat Xpert substudy entry to 3-month visit”.

 ********************

***5. Discussion:***

- *Although the authors note that their population was not limited to symptomatic individuals as a potential weakness, they do not explicitly state that an asymptomatic population is likely to have paucibacillary or subclinical disease, for which Xpert would not have good sensitivity. The SA algorithm for TB investigation starts with a positive symptom screen, not merely for being HIV positive with a low CD4 despite having no symptoms. So, these findings are not necessarily generalizeable to actual practice.*

**Response: **We do discuss the role of paucibacillary disease in the first paragraph of our discussion, and when describing study limitations but the text has been amended to include your recommendation to make a more explicit statement. We agree that current algorithms for TB start with symptom screening, but the need to identify active TB among asymptomatic HIV positive people is increasingly recognised, and our results have relevance to these efforts. We have added a sentence to the discussion to make this point.

The text reads as follows:

 We limited our study to HIV-positive individuals at highest risk of active TB, i.e. those who were WHO tool positive with CD4<200x10 ^6^/l, or pre-ART with CD4<200x10 ^6^/l, or newly diagnosed, **of whom some were asymptomatic** **,** to minimise unnecessary testing of individuals at lower risk of active TB. The low yield from repeat Xpert in those with negative initial Xpert is likely due to paucibacillary **or subclinical disease which is more likely in asymptomatic individuals** **,** or extrapulmonary disease. These TB diagnoses may be better identified by alternative diagnostic modalities, such as chest radiography.

 Our study has some limitations. In the parent XPHACTOR study, Xpert testing was prioritised in people with BMI<18.5kg/m ^2^ or CD4<100, those newly diagnosed HIV-positive or pre-ART with CD4<200, as well as those with TB symptoms. Thus the population in this substudy did not all have classic “TB symptoms” at the time of collection of either initial or repeat sputum samples for testing with Xpert. **These individuals may have had paucibacillary or subclinical disease which would not have been detected by Xpert .** However, TB prevalence in our substudy population was high, and we anticipate our results to be relevant at least to these high-risk groups. **Our results are also relevant to efforts to improve TB case finding among asymptomatic HIV-positive people**.

- *I would also note that the prevalence of clinical diagnosis is not necessarily generalizeable. Its unclear the level of care that was given to these participants- was this routine care available in a primary public health clinic, or is this specialized care as part of a research study? These details could help the reader evaluate how generalizeable the findings are.*

**Response:** All decisions regarding initiation of TB treatment were made by the routine care provider, and patients attended their routine care appointments at which they may have had additional investigations (including Xpert and mycobacterial culture) arranged by the clinic. We reviewed clinic records at the end of the study to obtain information regarding additional TB investigations and TB diagnoses made in routine care. This has been made clearer in the methods section as detailed above.

 As part of the research study additional Xpert testing was undertaken in accordance with the XPHACTOR study algorithm, and at the 3-month visit blood and sputum samples were taken for mycobacterial culture. As already described in the methods section our research staff facilitated the Xpert-negative algorithm when participants attended for Xpert result review, which should have been undertaken routinely for symptomatic individuals. Therefore these investigations are likely to have been initiated faster than in a routine setting. The research study is likely to identified both more confirmed TB and clinical TB diagnoses. We do refer to our optimised implementation of TB diagnostic algorithms when discussing the strengths of the study which should enable the reader to evaluate how generalizeable the findings are.

 ********************

***6. Conclusions:***

- *The reference to the "Xpert negative pathway" is vague - authors should be specific about what kinds of followup are most useful. Authors should also include something about LAM.*

**Response:** We have removed the LAM data from this manuscript. We have amended the text as per your recommendation. The text reads as follows:

 Amongst ambulatory HIV-positive individuals at high risk of active TB, if an initial Xpert is negative, the Xpert-negative pathway **(chest radiograph, mycobacterial culture on sputum, if appropriate antibiotic trial )** should be implemented and there should be a low threshold for investigating those who remain at high risk using all clinically appropriate diagnostic modalities.
